# Hydrogel-Based Biomaterial as a Scaffold for Gingival Regeneration: A Systematic Review of In Vitro Studies

**DOI:** 10.3390/polym15122591

**Published:** 2023-06-06

**Authors:** Dimas Ilham Hutomo, Lisa Amir, Dewi Fatma Suniarti, Endang Winiati Bachtiar, Yuniarti Soeroso

**Affiliations:** 1 Department of Periodontology, Faculty of Dentistry, Universitas Indonesia, Jakarta 10430, Indonesia; dimas.hutomo@ui.ac.id (D.I.H.); yuniarti@ui.ac.id (Y.S.); 2 Department of Oral Biology, Faculty of Dentistry, Universitas Indonesia, Jakarta 10430, Indonesia; dewi.fatma@ui.ac.id (D.F.S.); endang04@ui.ac.id (E.W.B.)

**Keywords:** hydrogel, gingival tissue regeneration, in vitro studies, systematic review

## Abstract

Background: Hydrogel is considered a promising scaffold biomaterial for gingival regeneration. In vitro experiments were carried out to test new potential biomaterials for future clinical practice. The systematic review of such in vitro studies could synthesize evidence of the characteristics of the developing biomaterials. This systematic review aimed to identify and synthesize in vitro studies that assessed the hydrogel scaffold for gingival regeneration. Methods: Data on experimental studies on the physical and biological properties of hydrogel were synthesized. A systematic review of the PubMed, Embase, ScienceDirect, and Scopus databases was conducted according to the Preferred Reporting System for Systematic Reviews and Meta-Analyses (PRISMA) 2020 statement guidelines. In total, 12 original articles on the physical and biological properties of hydrogels for gingival regeneration, published in the last 10 years, were identified. Results: One study only performed physical property analyses, two studies only performed biological property analyses, and nine studies performed both physical and biological property analyses. The incorporation of various natural polymers such as collagen, chitosan, and hyaluronic acids improved the biomaterial characteristics. The use of synthetic polymers faced some drawbacks in their physical and biological properties. Peptides, such as growth factors and arginine–glycine–aspartic acid (RGD), can be used to enhance cell adhesion and migration. Based on the available primary studies, all studies successfully present the potential of hydrogel characteristics in vitro and highlight the essential biomaterial properties for future periodontal regenerative treatment.

## 1. Introduction

Gingival recession, thin gingival phenotype, and lack of keratinized tissue around natural teeth and dental implants are the most common gingival tissue problems which require soft tissue reconstruction treatment. To date, a coronally advanced flap in combination with a connective tissue graft is still considered as the gold standard treatment that results in a high success rate, high esthetic outcome, and long-term soft tissue stability [1,2]. However, this technique has some disadvantages, as it requires additional surgical area to obtain the donor, resulting in a longer surgical procedure, increased patient morbidity, prolonged intra- and post-operative bleeding, palatal sensory dysfunction, and infection [3,4,5]. The lack of available tissue for autografts has instigated researchers across the globe to find alternatives to autografts. Research in soft tissue regeneration conducted in the last two decades has addressed this widespread issue by developing and integrating highly biocompatible yet sensitive materials to obtain alternative biomaterial scaffolds that can substitute connective tissue grafts for soft tissue regeneration [6,7].

Scaffolds are defined as biomaterials with a three-dimensional solid porous structure that plays a role in the promotion of interactions between cells and biomaterials, adhesion of cells, and the deposition of ECM; allows suitable transport of gasses and nutrients that permits the survival, differentiation, and proliferation of cells; and triggers a minimal toxicity or inflammation degree in vivo [8,9]. Biophysical cues such as scaffold physical properties, degradation, and architectural morphology and biochemical cues for exploiting natural molecules and spatiotemporal delivery of biomolecules are important factors to consider in designing biomaterial scaffolds for tissue regeneration [10,11]. A scaffold should maintain its shape during application, be easy to handle, and not cause damage to the tissue [12].

Hydrogels are defined as an insoluble three-dimensional network of polymer matrices made from macromers, hydrophilic homopolymers, and copolymers [13]. Different types of hydrogels have been tested for their potential use in tissue engineering. The most used hydrogels are made of synthetic monomers such as poly-ethylene glycol (PEG), poly-vinyl alcohol (PVA), and poly-2-hydroxyethyl methacrylate (PHEMA) and natural monomers such as agarose, alginate, hyaluronic acid, fibrin, and collagen [14]. The first use of hydrogels in the biomedical field consisting of crosslinking 2-hydroxyethyl methacrylate was introduced by Wictherle and Lim in 1960 [15]. Hydrogels are hydrophilic polymer networks that are capable of imbibing large amounts of water to mimic the extracellular matrix (ECM) [16]. They act as a temporary matrix when they are employed in order to secure the proliferation of cells, the deposition of the ECM, and tissue ingrowth until the regeneration of the newly desired tissue is achieved [17].

**Figure 1:**
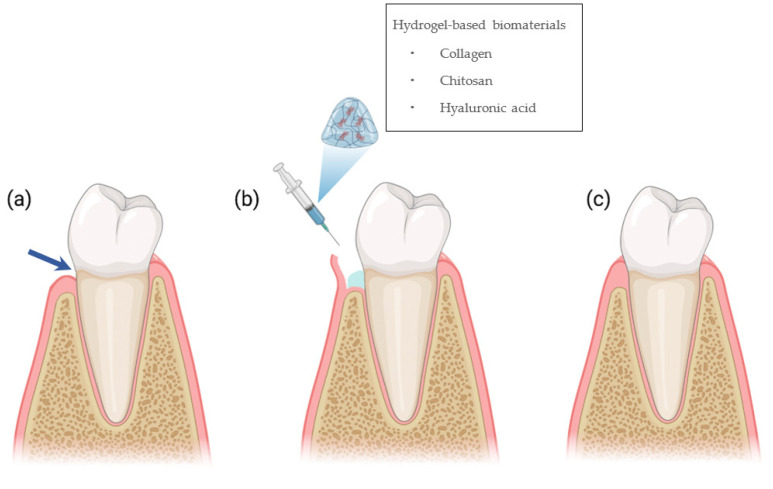
Schematic illustration of hydrogels for soft tissue regeneration. (**a**) Gingival recession indicated by blue arrow; (**b**) root coverage procedure followed by application of hydrogel-based scaffold; (**c**) healing of gingival tissue by the regeneration of the epithelial and the underlying connective tissue.

In recent years, hydrogels received attention in tissue engineering since they have a unique composition and structure resembling the natural ECM, serving as a desirable scaffold for cellular migration, proliferation, differentiation, neovascularization, and biomineralization [13,18]. The other advantages of hydrogels are their ability to absorb exudate from the wound surface and their ability to promote fibroblast proliferation, cell migration, and keratinization [15]. Furthermore, the shape adaptability characteristics of hydrogels for minimally invasive procedures make these biomolecules attractive as a scaffold biomaterial for tissue engineering purposes, including for oral tissue regeneration [18,19,20]. Studies have demonstrated the potential of hydrogels for the regeneration of dentin and dental pulp and tooth-supporting structures of periodontal tissues. The goal is to reconstruct the architecture and function of dental tissues and periodontal tissues including gingiva, periodontal ligament, cementum, and alveolar bone [13] (Figure 1).

Gingival tissues are the part of the oral epithelium that covers the alveolar bone of mandibular and maxillary bone. Numerous reports on the physical and biological properties of hydrogels demonstrated the increasing interest in this polymer matrix for its potential use in gingival tissue regeneration. Before its therapeutic use in human tissue, in vitro experiments were an important approach to understand the physical properties and biological properties of the biomaterials. The present systematic review aimed to identify and synthesize in vitro studies on hydrogels as biomaterial scaffolds for gingival regeneration based on their physical and biological properties. Systematic reviews of in vitro studies present an opportunity to synthesize evidence from numerous studies that address the same topic as new approaches for clinical translation.

## 2. Materials and Methods

The review protocol of this study was registered to the Open Science Framework Database (Registration DOI https://doi.org/10.17605/OSF.IO/ZP9DT), accessed on 30 April 2023.

### 2.1. Search Strategy

**Figure 2:**
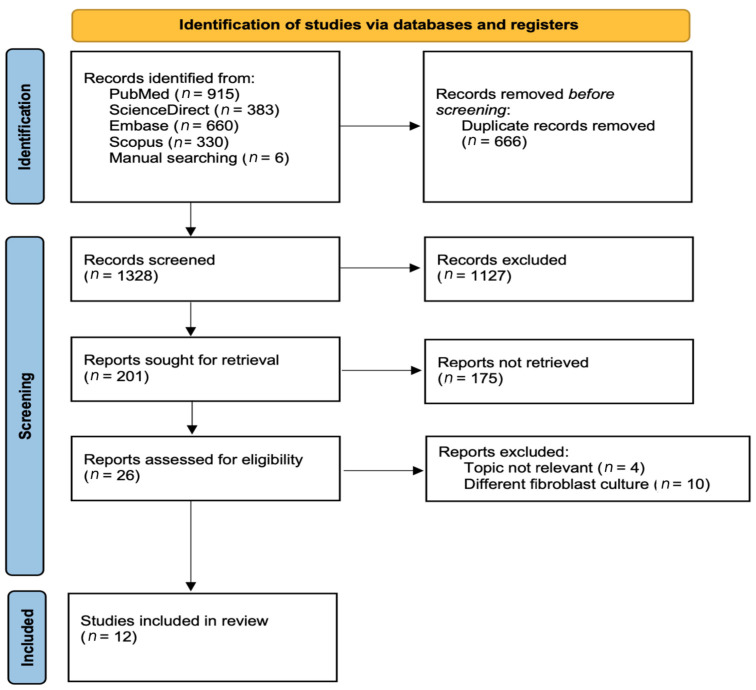
PRISMA flowchart for the screening protocol.

The review process followed the Preferred Reporting System for Systematic Reviews and Meta-Analyses (PRISMA) 2020 statement guidelines to review the physical and biological properties of hydrogels for gingival tissue regeneration (Figure 2). The research question was formulated using the population, intervention, comparison, and outcome (PICO) approach. Full-text papers written in English from PubMed, Embase, ScienceDirect, and Scopus data and a manual search of publications in the last 10 years were used (February 2012–February 2022). The search strategies, including the combination of keywords and the number of articles retrieved, are described in Table 1.

### 2.2. Eligibility Criteria of the Articles

Two reviewers (D.I.H and L.R.A) independently searched the titles and abstracts from electronic databases. Full reports were retrieved and examined independently in duplicate, and any disagreement was resolved with a third reviewer (D.F.S) if necessary to obtain relevant data for data analyses. The articles were selected for full-text reading and were assessed by individual reviews independently to obtain relevant data for qualitative review.

### 2.3. Inclusion and Exclusion Criteria

Full-text articles were considered eligible if they satisfied the following criteria: (1) in vitro experimental setup on primary or cell line of gingival fibroblast culture, (2) studies that analyzed the physical and/or biological properties of hydrogel. Meanwhile, as the current systematic review focused on the physical and biological properties of hydrogels, the exclusion criteria were as follows: (1) experiments other than on primary or cell line of gingival fibroblast culture, (2) clinical or randomized control trials or case reports or case studies, (3) narrative reviews or meta-analyses, (3) no abstract presented, and (4) duplicate publications.

### 2.4. Quality Assessment

To analyze the quality of the included studies, we used the clinical experimental checklist for non-randomized experimental studies by the Joanna Briggs Institute. The checklists were modified based on the research questions and PICOS structure [21]. The quality of each study was assessed using the nine questions presented in Table 2.

## 3. Results

### 3.1. Selection of Studies

The electronic and manual searches found 1328 articles of potential relevance after the removal of duplicates. Title and abstract screening were performed to meet the inclusion criteria. In total, 39 studies were fully analyzed to precisely check for the exclusion criteria, of which 27 articles were excluded. Finally, 12 articles were identified and included in this review. From the 12 included articles, 1 study performed a physical property analysis, 2 studies performed a biological property analysis, and 9 studies performed both physical and biological property analyses.

### 3.2. Characteristics of Studies

The selection of biomaterial combinations is an important consideration for the formulation of hydrogel-based scaffolds. Table 3 presents the 12 articles included in this review, which used 13 different biomaterials. The most common biomaterial used was collagen (five articles), followed by chitosan (three articles) and hyaluronic acid, PEG, and fibrin (two articles).

As biomaterial scaffolds, including hydrogel, are used for in situ tissue regeneration, the scaffold should have the ideal physical and biological properties before its application to human tissue. Of the 12 articles, we categorized the selected articles into two categories: (i) 9 articles that studied the physical properties of hydrogel and (ii) 10 articles that studied the biological properties of hydrogel in vitro.

### 3.3. Result of Studies Included

#### 3.3.1. Physical Property Analyses

Hydrogels are hydrated systems that consist of crosslinked hydrophilic units forming compact and stable polymer networks [34]. The fabrication of the hydrogels mostly involves physical or chemical crosslinking methods to obtain stable polymer networks [35]. All studies in this review performed a chemical crosslinking process to synthesize the hydrogels. The most popular chemical crosslinking techniques are complementing the group’s chemical reaction, high-energy radiation, free radical polymerization, and enzyme-mediated crosslinking [36]. The chemical crosslinking method produces hydrogels with higher mechanical properties and stability compared to physical crosslinking [37]. Increasing crosslinking density improved the stiffness, toughness, and degradation time due to a relatively more stable structure and more elasticity [38].

Of the nine studies that performed a physical analysis of the hydrogel properties, five studies analyzed the characteristics and swelling ratio. Scaffolds for tissue engineering initially fill a space in tissue and serve as a temporary matrix for newly regenerated tissue. Physical properties such as gel formation, mechanical characteristics, morphology, and biodegradation behavior of the biomaterials are important to the success of the scaffold [39]. Table 4 presents the biophysical characteristics of hydrogels designed for gingival tissue regeneration. Pores are important for cell proliferation and for nutrient and gas transfer while maintaining good mechanical properties of the scaffold [40]. Fibroblasts showed optimal cell proliferation in scaffolds with a pore size range of 50–160 μm and 86% porosity [41,42]. Hydrogels composed of combinations of PEG DVO, keratin fibrin, collagen alginate fibrin, collagen chitosan glycerol, and PVA collagen showed suitable pore sizes for fibroblast ingrowth [22,26,30,31,33].

#### 3.3.2. Biological Property Analyses

Figure 1 shows the 12 studies included in this systematic review. Overall, 10 studies produced hydrogel formulations using natural polymers (Col, CS, and HyA) as the biomaterials (Table 5). Collagen originates from the tissues of humans or any other species, has special physicochemical properties and architectural features, and is the most widely used natural polymer in biomedical fields [23,30,31,32,33,43]. It has the ability to promote cell adhesion, chemotaxis, homeostasis, physical degradation, and low toxicity, as well as induce the stimulation of fibroblasts’ DNA [42,43]. The characteristics of collagen were improved in combination with other polymers such as chitosan, gelatin, alginate, and PVA; growth factors; or bioparticles or by modifying its functional chain in the crosslinking process [30,31,33,44].

All studies included in this review performed a cytotoxicity assay, as toxicity screening is mandatory in the development of new biomaterials for pharmaceutical safety and highlights a crucial step in formulating scaffolds [45]. However, the lack of bioactive components is still a major challenge for tissue engineering as the cells cannot proliferate, differentiate, or migrate. The chemical modification allows the incorporation of natural scaffolds or bioactive molecules to increase its biological capability to ensure cell biology performance similar to the native environment [46].

## 4. Discussion

The trend of the development of biomaterial scaffolds in the periodontology field has increased in the last decade. Tissue engineering scaffolds provide a substitute for autografts for gingival tissue regeneration. Scaffolds interact with human tissue through their physical and biological properties by altering tissue microenvironments via modulating the host immune system and controlling the kinetics and healing capabilities of endogenous cells [47]. This systematic review has evidenced the biophysical and biological properties of various hydrogel formulations in vitro.

Based on origin, hydrogels can be classified as natural, synthetic, or a combination of both. Natural polymers are derived from biological materials present in nature that are extracted through physical or chemical methods. Instead, synthetic hydrogels are produced from synthetic polymers such as plastics, elastomers, and synthetic fibers. Both natural and synthetic hydrogels are developed through physical or chemical crosslinking, and they can be combined to improve their physical characteristics [44,48,49]. Natural polymers show good biological properties since they have physical and biological characteristics recognized in human tissue, while synthetic polymers have greater control of the characteristics and properties for tissue regeneration [13,50,51,52]. The structure of synthetic polymers can be modified to improve their biological performance. Hence, to improve hydrogel properties, it is applicable to combine natural polymers with synthetic polymers [53]. Pure PVA is a super-hydrophilic polymer that has poor cell adhesion properties due to its low affinity to protein. To overcome this limitation, physical modifications, such as extracellular matrix coating and air plasma treatment, have been shown to increase hydrogel surface roughness and have a positive effect on the cytoskeleton arrangement to promote cell attachment [54].

Ideally, scaffolds should have mechanical properties consistent with the desired tissue formed that are strong enough to facilitate good handling during implantation [55]. Due to their high water content, porosity, and mechanical tunability, hydrogels are particularly attractive as extracellular matrices to stimulate the regeneration of soft tissue [56]. The elastic characteristic of hydrogels resembles that of rubber, and existing theories of rubber elasticity are in line with hydrogel deformation during swelling [57]. The increase in pore size permits cells to avail the maximum internal surface of the scaffolds and facilitates cell infiltration into the scaffolds [29]. Six studies have demonstrated a high swelling rate of hydrogels [26,28,29,30,31,33]. The high swelling rate exhibits the water-absorbing capability of hydrogels making a moist environment for tissue engineering [58]. The swelling rate of hydrogels can be regulated by the mixture concentration. Montalbano and Rosdiani demonstrated low collagen concentration in the hydrogel mixture resulting in a decrease in swelling ratio [30,31]. Collagen has many hydrophilic groups to absorb water from its surroundings [31]. The swelling ratio is a parameter that can be used to examine the increase in a hydrogel’s weight due to water absorption, which could alter the cell growth on the hydrogel scaffold. A swelling ratio that is too fast may cause hydrogel network collapse before the desired new tissue has formed, whereas a swelling ratio that is too slow may inhibit the growth of cells inside the scaffold, thus preventing tissue formation within the scaffold [59].

Hydrogels must have appropriate mechanical and biological properties consistent with the desired tissue formed. They must support tissue growth and proliferation without causing toxic reactions in the cells. The structure of the hydrogels must be compatible with human cells and tissue without causing toxicity or altered immune reactions in the host after it degrades [60,61]. Previously, it was reported that most hydrogel mixtures have good biocompatibility due to the elastic and soft nature of the hydrogels that minimizes their irritability toward cells [36]. The existence of synthetic polymers in hydrogels has shown lower cell viability [32,33]. Synthetic polymers were reported to be less biocompatible compared to natural polymers, as they have no bioactive capacity [62]. The biofunctionalization of synthetic polymer structures was reported to increase scaffold–cell or scaffold–tissue interactions. Synthetic polymers can also be mixed with natural polymers or growth factors [63].

The extracellular matrix is a bioactive scaffold that contains many natural polymers that have the capacity to promote various types of tissue-specific cues, giving biological cues to the microenvironment surrounding the scaffold and ultimately promoting tissue regeneration [64]. Growth factors (GFs) are blood-derived peptide factors that direct inflammatory cells to migrate onto the wound, attract fibroblasts, and stimulate cell proliferation [65]. The addition of GFs as bioactive molecules into a hydrogel scaffold is a strategy to enhance the healing process [66]. For example, the incorporation of TGFβ-1 into a collagen hydrogel was shown to enhance fibroblast proliferation [23]. Gingival fibroblasts differentiate to myofibroblasts in response to the presence of TGFβ-1 in a stiff collagen substrate [67]. Myofibroblasts are a contractile phenotype of fibroblasts due to the upregulation of α-smooth muscle actin and matrix metalloproteinase [22]. They exhibit reinforced adhesion to the ECM and increased ECM remodeling [68]. Even though GFs are useful in designing a scaffold, several drawbacks of growth factor application in tissue regeneration have been reported, such as their short half-life after being delivered in vivo, poor stability, and systemic toxicity due to over-release [69,70,71]. Studies have shown hydrogels’ property of absorbing a large number of tissue exudates at the wound site to maintain a moist environment. This condition is favorable for growth factor release and subsequently promotes cell proliferation and differentiation to obtain rapid wound healing.

Gelation kinetics play a role in hydrogel delivery. Mechanisms of gel formation dictate how cells and molecules are integrated into the scaffold and how that scaffold is delivered [39]. Injectable hydrogels can be formed as a free-flowing solution that can be transformed into a semi-solid form under certain circumstances [72]. Thermo-responsive hydrogels are supramolecular hydrogels that undergo a gelation process via hydrophobic interaction. Hydrogels can undergo sol–gel phase transition because they consist of amphiphilic polymers with hydrophilic and hydrophobic components [73]. Hydrogels can undergo a gelation process from room temperature to body temperature (range of 25–37 °C), which is beneficial for their application in tissue engineering [58]. Collagen/alginate/fibrin hydrogels showed sol-phase transition at a temperature of 37 °C [30]. Slow gel formation results in poor adaptation and network formation, altering the cell encapsulation as the therapeutics may flow away from the targeted site. Furthermore, fast gel formation makes it difficult to inject in the targeted site [74,75].

The biodegradable property of biomaterials resembles an extracellular matrix and promotes tissue regeneration [34]. The biodegradation of hydrogels occurs based on several mechanisms, such as hydrolysis, photolysis, separation, or combinations thereof [76]. Scaffold degradation is mainly a chemical process, but it can be determined by physical stimuli to influence cell function and behavior. Degradation of the scaffold is followed by a decrease in its stiffness and is controlled by different polymer concentrations [30,77]. Longer hydrogel degradation occurred when the collagen concentration was reduced due to the presence of collagenase in the tissue host [30]. Ideally, the scaffold degradation rate is parallel to the rate of new tissue regeneration.

Fibroblasts have been regularly used to examine scaffold biocompatibility for soft tissue engineering and are the most abundant resident cells in the periodontium [22,78,79]. Approximately 2.10^8^ fibroblasts are estimated per 1 cm^3^ of connective tissue, equal to 5% volume. The goal of designing a biomaterial scaffold is to provide a temporary framework for the migration and attachment of fibroblasts from areas adjacent to the wound [65,80]. In soft tissue regeneration, the first step is gingival fibroblast migration to the scaffold and closing the dehiscence wound [81]. A recent study demonstrated that the addition of HyA to chitosan hydrogels and HyA showed higher fibroblast migration into the scaffold compared to chitosan alone [28]. The *CD44* receptors in fibroblast surfaces bind to HyA [82,83]. The ability to promote cell adhesion is also an important hydrogel property. The tripeptide arginine–glycine–aspartic acid (RGD) motif is a well-known general recognition motif acting via cell surface integrin receptions and is present in various extracellular matrix proteins [84]. The RGD peptide is commonly used to improve cell adhesion properties [84,85]. Cells seeded on hydrogels containing RGD showed good cell morphology and a greater number of cells compared to the non-RGD hydrogels [27]. The incorporation of the RGD peptide is useful for promoting the cell adhesion of synthetic materials [38]. RGD binds to several surface receptors due to the presence of integrin in the cell membranes. To date, 24 integrins have been identified to bind with ECM proteins containing RGD peptide sequences [86]. 

A mixture of gelatin and methacryloyl showed a decrease in cell viability because the high-density scaffold network reduced its porosity and, therefore, the transport of cell nutrients [32,87]. A similar result was found using PVA as the combination [33]. PVA has very low interactions with protein, resulting in less cell deposition on the scaffold surface. The residual charge of this polymer caused a disruption in the cell membrane [88]. Furthermore, any initiators, crosslinkers, or other additives used in hydrogels must not cause cellular toxicity. The polymerization process of hydrogels should not cause increased temperatures that could result in thermal necrosis on the tissue host [38]. PVA/collagen (50:50) hydrogels induced the differentiation of fibroblasts to form pseudopods [33]. Pseudopodia are essential for cell movement since they determine the trajectory, direction, and speed of cell migration [89]. In contrast, GelMA hydrogels failed to reveal fibroblast contraction after hydrogel implantation; this may alleviate the proliferation capacity of fibroblasts [32]. The migration and proliferation of fibroblasts at the defect sites lead to collagen deposition [90]. Collagen is a major protein secreted by fibroblasts in gingival ECM that serves as a structural scaffold in tissues [91,92]. The ultimate goal in tissue regeneration is to restore the integrity of the ECM structure and collagen networks under which the physiological regeneration occurs [93].

Although the majority of hydrogels are currently still under investigation, in vitro studies as well as numerous in vivo studies reported promising results that may lead to clinical studies. In vivo biocompatibility of hydrogels was observed, confirming the non-toxic nature of the biomaterials toward normal tissues [94]. Future research development of hydrogels as biomaterial scaffolds for gingival regeneration is promising. For example, the development of 3D printing technologies in tissue engineering has been increasing over the last decade [95,96]. This technology is able to produce an individualized scaffold to mimic tissue behavior with precise geometry matching the defects [97]. Peng et al. successfully constructed a 3D printing scaffold consisting of acellular dermal matrix/gelatin-sodium alginate (ADM/A/G) with living gingival fibroblasts which was able to promote cell proliferation and increase the expression of specific tissue biomarkers in vitro [98].

### Strength and Limitations

This article presents a systematic review of the biophysical and biological properties of various hydrogels for gingival tissue regeneration. Although there are many reviews that have focused on hydrogel use in tissue engineering, we still found a lack of articles that have focused on hydrogel use in gingival tissue regeneration. One limitation of this study is that few previous studies have analyzed hydrogels as gingival scaffolds, and some authors did not reveal the uniform analysis performed in their studies. Further research is needed to develop hydrogel scaffolds with tunable mechanical properties that induce the regenerative process through the stimulation of cell function, the matching of the degradation rate and the physiological remodeling process, and functionalization to enhance cell interaction. Hence, extensive in vivo and clinical trial studies should be further investigated for the application of hydrogel scaffolds in gingival tissue engineering, which is also one of the main directions of biomaterial research and development in the future.

## 5. Conclusions

The trend of substitute biomaterial research in the periodontology field, especially for gingival regeneration, is increasing, as it potentially reduces the use of autografts and thereby reduces patient morbidity. Hydrogels are potential biomaterials to be used as a substitute for autografts. In this systematic review, the authors summarized research on hydrogels as scaffold biomaterials in vitro. Natural hydrogels are still the most popular polymers in designing hydrogels. Further research is necessary to determine which composition suits clinical uses in daily practice following the parameters that are discussed in this systematic review.

## Figures and Tables

**Table 1:** Databases and research method.

Database	Search Strategy
PubMed	(hydrogel) AND (injectable)) OR (gingival [MeSH Terms])) OR (periodontal [MeSH Terms])) AND (regeneration [MeSH Terms])) AND (natural polymer)) OR (synthetic polymer)) AND (in vitro [MeSH Terms])
Science Direct	hydrogel OR injectable AND natural polymer OR synthetic polymer AND gingival regeneration OR periodontal regeneration AND in vitro
Embase and Scopus	(hydrogel:ti OR injectable:ti) AND (‘natural polymers’ OR (‘natural’/exp OR natural) AND (‘polymers’/exp OR polymers)) OR ‘hyaluronic acid’/exp OR ‘hyaluronic acid’ OR (hyaluronic AND (‘acid’/exp OR acid)) OR collagen OR ‘chitosan’/exp OR chitosan OR ‘gelatin’/exp OR gelatin OR ‘cellulose’/exp OR cellulose OR ‘hyaluronan’/exp OR hyaluronan OR ‘agarose’/exp OR agarose OR ‘natural scaffold’ OR ((‘natural’/exp OR natural) AND (‘scaffold’/exp OR scaffold)) OR ‘synthetic polymer’/exp OR ‘synthetic polymer’ OR (synthetic AND (polymer’/exp OR polymer)) OR ‘polyester’/exp OR polyester OR ‘poly aci’ OR (poly AND lactic AND aciD) OR ‘poly glycolic acid’/exp OR ‘poly glycolic acid’ OR ‘plga’/exp OR plga OR ‘pga’/exp OR pga OR ‘peg’/exp OR peg OR (poly AND (‘ethylene glycol’/exp OR ‘ethylene glycol’ OR ((‘ethylene’/exp OR ethylene) AND (‘glycol’/exp OR alcohol)) |) OR (poly AND (‘vinyl alcohol’/exp OR ‘vinyl alcohol’ OR ((‘vinyl’/exp OR vinyl) AND (‘alcohol’/exp OR alcohol)) AND ‘gingival regeneration’ OR ‘gingival fibroblasts’ OR (gingival AND (‘regeneration’/exp OF regeneration))

**Table 2:** Quality assessment.

Authors	Joanna Briggs Institute Items									Raw Score and %	Risk
	X	Q2	Q3	Q4	Q5	Q6	Q7	Q8	Q9		
Cheung [22]	1	1	1	1	1	1	1	1	U	89%	Low
Choi [23]	1	1	1	1	1	1	1	1	U	89%	Low
Colangelo [24]	1	1	U	1	1	1	1	1	U	78%	Low
Cozens [25]	1	1	0	1	1	1	1	1	U	78%	Low
Kang [26]	1	1	1	0	1	1	1	1	U	78%	Low
Laird [27]	1	1	1	1	1	1	1	1	U	89%	Low
Miranda [28]	1	1	1	1	1	1	1	1	U	89%	Low
Moatary [29]	1	1	1	1	1	1	1	1	U	89%	Low
Montalbano [30]	1	1	1	1	1	1	1	1	U	89%	Low
Rosdiani [31]	1	1	0	1	1	1	1	1	U	78%	Low
Tabatabaei [32]	1	1	1	1	1	1	1	1	U	89%	Low
Zhou [33]	1	1	0	0	1	1	1	1	U	67%	Moderate

Q1: Were experimental conditions identical across study groups? Q2: Were all measured outcomes reported? Q3: Was there a control group? Q4: Was the rationale for hydrogel mixture concentration used explained? Q5: Were there multiple measurements of the outcome both before and after the intervention/exposure? Q6: Was follow-up complete and, if not, were differences between groups in terms of their follow-up adequately described and analyzed? Q7: Were the outcomes of participants included in any comparisons measured in the same way? Q8: Were the quantitative data mentioned? Q9: Were the experiments performed in replicates?

**Table 3:** Characteristics of the included studies.

No.	Author	Biomaterial	Crosslinking Method	Fabrication
1	Cheung [22]	DVO, PEG	Chemical	D-PHI flat films were generated using a divinyl oligomer (DVO); PEG was used as porogen
2	Choi [23]	Collagen, growth factor (TGF-b1), nanoparticles (gold, TaO, dextran, or ferritin)	Chemical	Collagen type I from rat tail tendons was loaded with either TGF-b1 or other nanoparticles such as gold, TaO, dextran, or ferritin
3	Colangelo [24]	PN, HyA	Chemical	PN extraction from salmon trout gonads mixed with HyA
4	Cozens [25]	PAA, Tyr, Cys, BA, BP	Chemical	Coupling amine to PAA using 4-(4,6-dime- thoxy-1,3,5-triazin-2-yl)-4-methylmorpholinium chloride (DMTMM) and then crosslinking with Tyr/Cys/BA/BP
5	Kang [26]	KRT, FIB	Chemical	Keratin from human hair mixed with fibrinogen from human plasma
6	Laird [27]	PEG, gyrase B (with/without RGD motifs), coumermycin, novobiocin	Chemical	PEG added to mixture of Novobiocin (antibiotic) or Coumermycin (dimeric form of novobiocin) and GyrB with/without RGD motifs (protein expressed by *Eschericia coli*)
7	Miranda [28]	Chitosan, HyA	Chemical	Medium-molecular-weight chitosan was succinylated, and HyA oxides were mixed
8	Moatary [29]	Nano diopside, b-chitin, chitosan	Chemical	Nano diopside ceramic was prepared by a modified sol–gel method then added to sulfate derivative of chitin and chitosan
9	Montalbano [30]	Collagen, alginate, fibrin	Chemical	Collagen type I from calf skin, low-viscosity alginate from brown algae, fibrinogen from bovine plasma
10	Tabatabaei [32]	Collagen, GelMA	Chemical	Cell-embedded collagen and collagen-embedded gelatin methacryloyl (GelMA)
11	Rosdiani [31]	Collagen, chitosan, glycerol	Chemical	Collagen and chitosan powders from tissue banks were dissolved into the solvents, and then glycerol was added
12	Zhou [33]	PVA, collagen	Chemical	Tilapia collagen type I mixed with PVA

PN = polynucleotides, HyA = hyaluronic acid, Cys = cystine, Tyr = tyramine, BA = bromine moieties, BP = boronic acid moieties, KRT = keratin, CS = chitosan, FIB = fibrin/fibrinogen, Col = collagen, TGF = transforming growth factor, TaO = tantalum oxide, PEG = polyethylene glycol, DVO = divinyl oligomer.

**Table 4:** Physical properties of hydrogels.

No.	Authors	Type of Scaffold	Physical Property Analyses	Main Findings
1	Cheung et al. [22]	- PEG + DVO	- Characterization	- Size of pores: 30–250 μm.
2	Choi et al. [23]	- Collagen + TGFb-1 - Collagen + gold - Collagen + TaO - Collagen + dextran - Collagen + ferritin	- Characterization	- Hydrogel loaded with gold or TaO had smallest pore size, while hydrogel loaded with dextran showed largest pore size.
3	Cozens et al. [25]	- PAA + Cys - PAA + Tyr - PAA + BP - PAA + BA	- Morphology - Rheology - Hydrogel bonding	- PAA-BP and PAA-Cys showed the highest adhesion strength and energy density for porcine keratinized gingiva. - PAA-BP produced high moduli and elasticity, resulting in strong adhesion to tissues. - PAA-Tyr and PAA-BA displayed weak mechanical properties.
4	Kang et al. [26]	- Keratin + Fibrinogen (in different concentration)	- Morphology - Rheology - Injectable performances - Swelling capacity - Biodegradation	- The porosity and viscosity of keratin–fibrinogen hydrogel were improved by increasing the molar ratio of KRT to FIB so the hydrogel could be injected through needle extrusion. - The molar ratio of KRT:FIB = 3:1 was the most suitable for the biomaterial scaffold with a highly porous structure (10–100 mm). - The 3:1 mol ratio of KRT:FIB had a swelling ratio almost 6 times higher than that of FIB.
5	Miranda et al. [28]	- Chitosan - HyA - Chitosan + HyA (in different concentration)	- Characterization - Swelling capacity	- HyA and CS-HA hydrogels showed irregular structures with higher pore sizes compared to CS hydrogels. - HyA and CS scaffolds showed higher swelling ratios.
6	Moatary et al. [29]	- Chitin hydrogel +/chitosan/nano diopside	- Characterization - Porosity - Water uptake - Mechanical - Swelling capacity - Degradation	- Chitin/chitosan/nano diopside had homogeneous porous structures, suitable for scaffolds. - They were to retain water, so the internal surface area was increased. - Increased mechanical stability. - Addition of nano diopside controlled the swelling rate and reduced the biodegradation rate.
7	Montalbano et al. [30]	- Collagen + Alginate + Fibrin	- Characterization - Gelation time - Swelling - Degradation - Rheology - Degradation	- Pore size: 40–120 μm. - Increased collagen concentration and less interconnected pores were observed, along with more stiffness and a decrease in the swelling ratio. - Gelation time happened at temperature of 37. - Completely dissolved in 3 h; degradation rate faster in low collagen concentration.
8	Rosdiani et al. [31]	- Collagen-Chitosan-Glycerol (in different concentration)	- Morphology - Mechanical strength - Swelling capacity	- The pore size obtained ranges between 102.4 and 143.5 μm, which is suitable for periodontal application. - Greater collagen concentration showed better mechanical strength but the lowest swelling capacity.
9	Zhou et al. [33]	- PVA + Col (in different concentration)	- Morphology - Swelling capacity	- PVA/Col (90:10) blended hydrogel exhibited a homogeneous dense surface layer, and small pores were observed at high magnifications; the addition of Col increased the porosity and pore size of the hydrogel and the swelling rate.

**Table 5:** Biological properties of hydrogels.

No.	Author	Type of Scaffold	HGF Cell Culture	Biological Property Analyses	Main Findings
1	Cheung et al. [22]	- PEG + DVO	ATCC	- Cytotoxicity - Cell proliferation - DNA mass quantification - Metabolic activity - Histology - Cell morphology - Protein measurement	- Experiments were performed in static and dynamic cell cultures. - HGF population remained viable and increased over 14 days of culture. - Metabolic activity increased significantly in the fourth week. - More cells were found in the inner regions of the scaffold in the dynamic culture, particularly on days 14 and 28. - Accumulation of Col I in the dynamic culture was greater than that in the static culture.
2	Choi et al. [23]	- Collagen + TGFb-1 - Collagen + gold - Collagen + TaO - Collagen + dextran - Collagen + ferritin	Primary HGF	- Cytotoxicity - Cell proliferation	- TGF-β1, in facilitating the recovery and proliferation of human gingival cells, was assessed when TGF-β1 was incorporated into and excreted from physiologically degraded collagen hydrogels. - TGF-β1 and TGF β1-carrying hydrogels showed larger amounts and metabolic activity of live cells. - Cells became attached and grew well due to the surface of collagen hydrogels.
3	Colangelo et al. [24]	- PN - HyA - PN + HyA	ATCC	- Cytotoxicity	- PN increased the assay signal in a visible and significant way; PN + HA failed to further increase cell growth as compared to PN alone.
4	Kang et al. [26]	- Keratin + Fibrinogen (in different concentration)	Primary HGF from ScienCell	- Cytotoxicity	- KFH exhibited much higher cell viabilities and supported cell proliferation. - KFH was more resistant to high-temperature and acidic conditions than FIB-H, protecting the integrity of its structure.
5	Laird et al. [27]	- PEG + gyrase B (with/without RGD motifs) + coumermycin, - PEG + gyrase B (with/without RGD motifs) + novobiocin	Primary HGF	- Cytotoxicity - Cell morphology	- Cells grown on hydrogels with RGD motifs showed normal morphology; meanwhile, cells exhibited unusual morphology indicating cell apoptosis on hydrogels without RGD motifs. - Hydrogels containing novobiocin exhibited a reduction in cell number.
6	Miranda et al. [28]	- Chitosan - HyA - Chitosan + HyA (in different concentration)	NIH3T3	- Cytotoxicity - Cell morphology	- An increase in cell viability was observed after the cells were seeded on the scaffold. - Migration rates of the cells were higher when they were seeded on the scaffold.
7	Moatary et al. [29]	- Chitin hydrogel +/chitosan/nano diopside	ESK-1	- Cytotoxicity - Cell attachment	- Chitin/chitosan/nano diopside composite scaffolds were cytocompatible and non-toxic to fibroblast cells. - Nanosurfaces had larger surface areas, which allowed the attachment of more cells.
8	Montalbano et al. [30]	- Collagen + Alginate + Fibrin	L929	- Cytotoxicity - DNA quantification - Metabolic activity - Cell morphology	- Cells were still viable with intact cell membranes. - No significant change in cell morphology was observed with changing collagen concentration. - Cells were proliferating; no significant differences in cell number and proliferation rate were observed.
9	Tabatabaei et al. [32]	- Gelatin methacryloyl (GelMA) - Collagen	Primary HGF	- Cytotoxicity - Morphology	- Cells were more likely to survive in collagen hydrogels. - GelMA hydrogels maintain cell initial shape and size without noticeable contraction.
10	Zhou et al. [33]	- PVA + Col (in different concentration)	HGFs #2620	- Cytotoxicity - Cell morphology	- Pure PVA showed lowest cell viability and cell adhesion; meanwhile, the highest was in PVA/Col (50:50). - On PVA/Col (100:0, 90:10, 70:30) blended hydrogels, HGFs appeared as round cells without pseudopods. HGFs with pseudopods were found in the scaffolds of the PVA/Col (50:50).

## Data Availability

Not applicable.
